# Supplementary material for: Association between genetic polymorphisms of the IL28B gene and leukomonocyte in Chinese hepatitis B virus-infected individuals
Source: PeerJ. 2017 Dec 19;5:e4149. doi: 10.7717/peerj.4149 (PMC5740955; doi:10.7717/peerj.4149)
Supplement: Supplemental Information 1 — Primers for PCR amplification and genotyping. [file peerj-05-4149-s001.doc]

Supplementary Table 1. Primers for PCR amplification and genotyping

| SNP | PCR primers | Genotyping probes |
| --- | --- | --- |
| rs12979860 | TCGTGCCTGTCGTGTACTGAAC  TATGTCAGCGCCCACAATTCC | TTTTTTTTTTTTTTTTGAACCAGGGAGCTCCCCGAAGGCGC  TTTTTTTTTTTTTTTTTTTGAACCAGGGAGCTCCCCGAAGGCGT  -P-GAACCAGGGTTGAATTGCACTCCGCTTTTTTTTTTTT-HEX- |
| rs8099917 | GTCTTGTATTTCACCTCCTGG  AATTGACGGGCCATCTGTTTC | TTTTTTTTTTTTTTTGTTTTCCTTTCTGTGAGCAATG  TTTTTTTTTTTTTTTTTTGTTTTCCTTTCTGTGAGCAATT  -P-TCACCCAAATTGGAACCATGCTGTATTTTTTTTT-FAM- |
| rs12980275 | GTATGATTCCCCCTACATGAG  ATTACAGGTCTGGTCCTAGTG | TTTTTTTTTTTTTTTTGAGAGAAGTCAAATTCCTAGAAACA  TTTTTTTTTTTTTTTTTTTGAGAGAAGTCAAATTCCTAGAAACG  -P-GACGTGTCTAAATATTTGCCGGGGTTTTTTTTTTTTT-FAM- |
